# Effects of Highway-Related Pollutant on the Groundwater Quality of Turfy Swamps in the Changbai Mountain Area

**DOI:** 10.3390/ijerph15081652

**Published:** 2018-08-03

**Authors:** Hong Wang, Lei Nie, Yan Xu, Chao Du, Tao Zhang, Yuzheng Wang

**Affiliations:** Construction Engineering College, Jilin University, Xi Min Zhu Street, Changchun 130026, Jilin, China; wanghong15@mails.jlu.edu.cn (H.W.); nielei@jlu.edu.cn (L.N.); duchao16@mails.jlu.edu.cn (C.D.); zhangtao16@mails.jlu.edu.cn (T.Z.); wangyz16@mails.jlu.edu.cn (Y.W.)

**Keywords:** turfy swamp, heavy metals, groundwater quality, highway transportation

## Abstract

Transportation activities such as fuel consumption, vehicle wear and road deicing can detrimentally affect the groundwater quality of fragile roadside wetland environments including. Nineteen parameters (Cu, Pb, Zn, Cd, Cr, Ni, Hg, As, pH, TDS, Ca^2+^, Mg^2+^, Na^+^, K^+^, SO_4_^2−^, Cl^−^, HCO_3_^−^, NO_3_^−^ and F^−^) were determined in groundwater samples from turfy swamps impacted by highway traffic from Jiangyuan (JY), Longquan (LQ), and Huangsongdian (HSD). Our results indicate that the metals Cu, Pb, Zn, Cr, Cd, the ions Na^+^, K^+^ and Cl^−^ in groundwater were negatively affected by highway transportation, and the maximum affected distance of these pollutants varied from 15 to 100 m. The content of most of these pollutants in roadside groundwater decreased exponentially with the distance from the highway, as did the heavy metal pollution index HPI and *C_d_*. The values of HPI and *C_d_* in these three sites ranged from 46.8 to 78.4 and −4.9 to −2.9, respectively. The low pollution levels of heavy metals are related to the strong adsorption capacity of turfy soil towards metals. In any case, road transport activities increased the Cu, Pb, Zn, Cr, Cd, Na^+^, K^+^ and Cl^−^ content in roadside groundwater in turfy swamp. With the increase of highway operation time, it will inevitably have a great influence on the groundwater quality of these wetlands. Therefore, the long-term monitoring is necessary to protect the sustainable development of turfy swamp.

## 1. Introduction

Environmental pollutants from traffic infrastructure are an important subject in ecological and environmental sciences, due to their toxic effects on the biosphere [1,2,3]. It is generally accepted that highway transportation activities such as the combustion of liquid fuels, vehicular component abrasion and the weathering of pavement materials can release fine particles containing Zn, Pb, Cd, Cu and Cr [4,5,6,7]. These particles containing heavy metals are deposited into soil, water and plant near the highway through dry and wet deposition [8]. In addition, some studies have reported that road-salting activities can increase the contents of Na^+^, K^+^, Cl^−^ and NO_3_^−^ in roadside groundwater and even cause severe Cl^−^ contamination [9,10,11,12]. These highway-related pollutants are persistent and non-degradable, and therefore can remain in the roadside environment for long periods of time. When their accumulations exceed a certain level, they will pose a threat to the microbiota, flora and fauna in turfy swamps [13].

Since the 1970s, concern about highway-related pollutants has spread beyond academia [14,15,16]. Studies of highway-related pollutants have focused on the identification of type of pollutants and their distribution influenced by distance from the roadway [17,18,19]. Zhang et al. [8] discovered that soil concentrations of Zn, Cd, Cu, As and Pb were the most affected by highway transport in the Tibetan plateau. Earon et al. [12] demonstrated that the metals Fe, Al, Zn, Mn, Pb, Ni, Cr, Cd and the ions Ca^2+^, SO_4_^2−^, K^+^, Na^+^, Mg^2+^, NO_3_^−^, and Cl^−^ in groundwater are significantly related to road transportation, decreasing exponentially as the distance from the roadside increases. In addition, many monitoring studies have demonstrated Pb, Cu, Cr, Cd and Zn pollution are severe in diverse roadside environments, including Beijing [4], Hong Kong [20], Mexico [21], and Yorkshire, England [22]. However, all these studies were mainly concentrated on roadside soil, with relatively little attention being paid to the effects of traffic pollution on groundwater. As far as we know, environmental pollutant levels in turfy swamp groundwater coming from highways have not been reported.

Wetland ecosystems have strong hydrological dependence, with the healthy water quality and hydrochemical characteristics being a determining factor in their sustainable development [23]. Turfy swamp is a unique natural ecosystem formed by interaction between water and land system [24]. Therefore, groundwater is extremely essential for survival of all living organisms in turfy swamps, which is sensitive to outside influences. Even small-scale human activities can result in significant environmental changes [25,26,27]. In the Changbai Mountain area, most of the turfy swamps are well preserved. However, increasing highway construction activities have caused many environmental problems in turfy swamps in recent decades. For example, Wang et al. [28] reported that metals Zn, Cr, Cu and Cd have reached moderate pollution in roadside soil in turfy swamp areas. However, the effect of highway on the heavy metal and hydrochemical characteristic of groundwater in turfy swamp is not clear. To formulate effective measures for turfy swamp protection, investigating the source and pollution states of highway-related pollutants in groundwater of turfy swamps is crucial.

This paper quantifies the impact of highway transport on groundwater in turfy swamps in the Changbai Mountain area from the two aspects of heavy metal and hydrochemical characteristics. Based on findings from a large-scale field investigation, we identify the types of highway-related pollutants in turfy swamp groundwater, and explore the relationships between highway-related pollutants concentrations and roadside distance, and lastly assess the heavy metal contamination levels of turfy swamp groundwater.

## 2. Materials and Methods

### 2.1. Site Description

**Figure 1:**
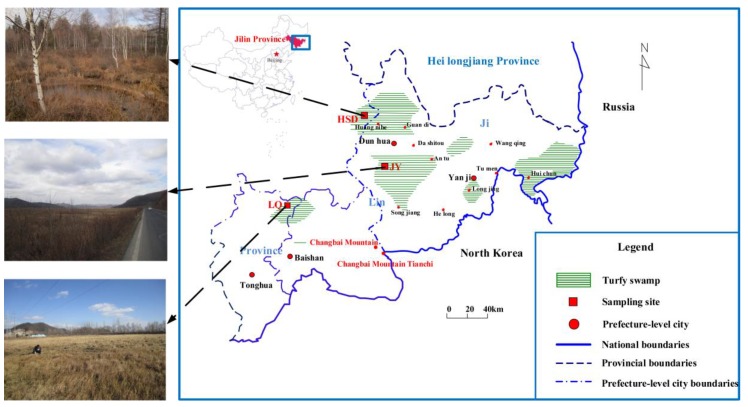
Study area and sampling sites in the Changbai Mountain area, Jilin Province.

This study was performed in the Changbai Mountain area, where a large variety of turfy swamps can be found. This region is the main headstream of the Mudan River and an important water conservation area in Jilin Province. Three sampling sites without any other confounding anthropogenic pollution sources were selected for collection of groundwater samples: Jiangyuan (JY, N43°7′, E128°1′), Longquan (LQ, N42°25′, E126°36′), and Huangsongdian (HSD, N43°39′, E127°39′) (Figure 1). At the JY site, the climate is cool and humid, with moderate illumination and abundant rainfall; the average annual temperature and precipitation are 2.6 °C and 632 mm. G201 highway with daily traffic volume greater than 2000 invades the edge of the turfy swamps. The climate characteristics at the LQ site are similar to JY site, with average annual temperature 3.7 °C and precipitation 947 mm; the S302 highway with daily traffic volume greater than 1500 invades the middle of the turfy swamp. For HSD, the average annual temperature and precipitation are 3.4 °C and 709 mm; G12 highway passes through the edge of the turfy swamp, with daily traffic volume greater than 1100. Regarding specific information about these three sites, readers may refer to Wang et al. [28].

### 2.2. Sampling and Experimental Methods

Groundwater samples were collected in July–August 2017 at distances of 5, 15, 50, and 200 m, from the highway, respectively at the three sites. Before sampling, three PVC tubes with a diameter of 160 mm were embedded in the soil at each distance, separated by 100 m, exposing 50 cm above ground and embedding 60 cm underground. Many holes with a diameter of 4 mm were drilled in the PVC tube wall to allow the groundwater to naturally infiltrate into the tube. In order to prevent sediment, branches and rainwater from entering the tubes PVC, the PVC outer wall was covered with gauze, and the tube end was covered with a plastic cloth fixed with a nylon rope. After groundwater samples were collected using the erect water collectors, the samples were filtered using a 0.45 μm filter membrane, and then transported to the laboratory at 2–5 °C in a cooler. Nineteen physical and chemical parameters, including Cu, Pb, Zn, Cd, Cr, Ni, Hg, As, pH, TDS, Ca^2+^, Mg^2+^, Na^+^, K^+^, SO_4_^2−^, Cl^−^, HCO_3_^−^, NO_3_^−^ and F^−^, were analyzed. The pH and total dissolved solids (TDS) were determined in the field using a portable measuring instrument (HI98195, HANNA, Padua, Italy). All samples were collected and stored according to the standard for detection of groundwater quality (DZ/T 0064.2-1993); the specific preservation and test methods are shown in Table 1. All samples were tested within 15 days after sampling at the Jilin University Testing Center.

### 2.3. Data Analysis Methods

A regression model was used to describe the relationship between the content of highway-related pollutants and the distance from the roadside using Origin 9.0 software (Origin Lab, Northampton, MA, USA). The high R^2^ values indicate that this model can well quantify the distribution characteristics of highway-related pollutants contents in groundwater.

To evaluate the pollution level of groundwater in turfy swamp, the heavy metal pollution index HPI and *C_d_* were calculated by the following formulas [29]:

(1) $$HPI=\frac{\sum_{i=1}^{n}W_{i}Q_{i}}{\sum_{i=1}^{n}W_{i}}$$

(2) $$Q_{i}=\sum_{i=1}^{n}\frac{\left(M_{i}(-)I_{i}\right)}{\left(S_{i}-I_{i}\right)}\times 100$$

where *Q_i_* and *W_i_* are the subindex and unit weight of the *i*-th element. *n* is the number of elements considered. *M_i_* is the measured value of the *i*th metal, *I_i_* and *S_i_* are ideal and standard values of the *i*th parameter, respectively. The sign (−) indicates the numerical difference of the two values, ignoring the algebraic sign. To calculate *W_i_* the groundwater class III standards (GB/T 14848-2017) were used for Cu, Pb, Zn, Cd, Cr, Ni, Hg and As.

The *C_d_* index indicates the relative contamination of different metals separately and manifests the combined effects of all metals, is calculated as follows:

(3) $$C_{d}=\sum_{i=1}^{n}C_{fi}$$

(4) $$C_{fi}=\frac{C_{Ai}}{C_{Ni}}-1$$

where *C_Ai_* and *C_Ni_* are the measured value and upper allowable value of *i*th metal (*N* refers to the normative value). *C_Ni_* is considered as the standard permissible value (*S_i_*) formerly introduced in HPI calculation.

The experimental data were subjected to statistical analysis using SPSS 21.0 software (SPSS Inc., Chicago, IL, USA), including the description analysis, K-S test, ANOVA, cluster analysis, factor analysis and Pearson correlation analysis. The ANOVA and LSD test were used to identify the significant differences of the measured parameters among different sites. The correlation analysis was used to explore the relationship between the measured parameters and distance from highway. The cluster analysis and factor analysis was performed to identify the interrelationship among these parameters in groundwater of turfy swamp. Euclidean distance and Ward’s method were used in cluster analysis, and the primary data were standardized using Z score.

## 3. Results and Discussion

### 3.1. Concentration of Metals and Hydrochemical Parameters in Groundwater

The basic statistical descriptions of heavy metals contents for different turfy swamp sites in roadside groundwater are summarized in Table 2. The maximum concentrations of all heavy metals in this area are lower than the drinking water quality of China (GB/T 14848-2017). The low contents of metals may be related to turfy soil with high organic matter content. The concentrations of Cu, Pb, Zn, Cr, Cd and Hg (58.3 μg L^−1^, 26.3 μg L^−1^, 114.2 μg L^−1^, 1.8 μg L^−1^, 5.4 μg L^−1^) at LQ turfy swamp are the highest among the three sites, while the Ni and As contents (8.9 μg L^−1^, 8.7 μg L^−1^) in JY are the highest. The ANOVA results indicated that the contents of most metals at the JY and LQ sites were significantly higher than those at the HSD one (*p* < 0.05). This finding is in accordance with the traffic volume in these three sites. The daily traffic volume at HSD is lower than that at JY and LQ site as previously reported by Wang et al. [28].

As shown in Table 3, the hydrochemical analysis results indicated that the mean concentrations of TDS (218.9 mg L^−1^), Ca^2+^ (29.3 mg L^−1^), Mg^2+^(5.8 mg L^−1^), Na^+^ (2.9 mg L^−1^), K^+^ (1.2 mg L^−1^), SO_4_^2−^ (11.6 mg L^−1^), Cl^−^ (5.5 mg L^−1^), HCO_3_^−^ (179.8 mg L^−1^), NO_3_^−^ (0.44 mg L^−1^), F^−^ (0.16 mg L^−1^) of the entire region were lower than the drinking water quality standards. The ACV% of these measured parameters followed the sequence K^+^ > Cr > Na^+^ > Cl^−^ > Pb > Cu > Zn > Cd > Mg^2+^ > pH > As > Ni > NO_3_^−^ > F^−^ > Hg > SO_4_^2−^ > Ca^2+^ > TDS > HCO_3_^−^ and the CV% of Cu, Pb, Zn, Cr, Cd, Na^+^, K^+^, Cl^−^ are significantly higher than those of the remaining parameters in the three sites. This result indicated that the concentrations of Cu, Pb, Zn, Cr, Cd, Na^+^, K^+^ and Cl^−^ varied greatly in different distances from the highway in turfy swamps, which leads us to speculate that concentrations of Cu, Pb, Zn, Cr, Cd, Na^+^, K^+^ and Cl^−^ in groundwater were most likely related to human activities along the highway in the turfy swamp areas.

### 3.2. Interrelationships among Measured Parameters in Groundwater in Turfy Swamp

**Figure 2:**
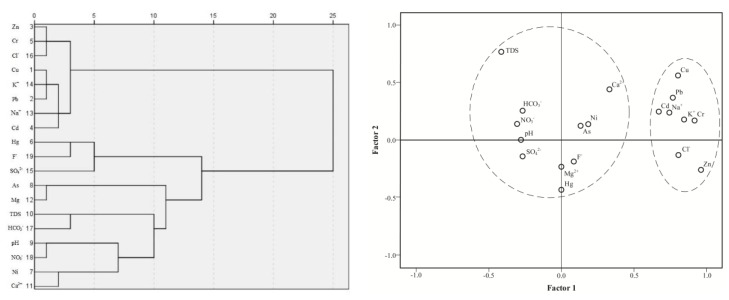
Cluster tree and scatter plots and of the 19 groundwater physicochemical indexes showing interrelationships among them.

Cluster analysis and factor analysis were performed on the 19 parameters determined in roadside groundwater to identify a shared source of these parameters in turfy swamps (Figure 2). As previously reported by Lee et al. [30] and Khan et al. [31], elements in the same group are expected to be from a common anthropogenic or natural source and a lower cluster distance indicates a more significant association. Two different cluster groups were presented at the three sites, Cu, Pb, Zn, Cr, Cd, Na^+^, K^+^ and Cl^−^ with a distance between 5 and 10 indicated that these parameters are from the same source. The remaining parameters are clustered at a high distance criterion between 10 and 15, possibly due to environmental heterogeneity of turfy swamp. 

The results of factor analysis indicated that Cu, Pb, Zn, Cr, Cd, Na^+^, K^+^ and Cl^−^ in groundwater are significantly related to each other. A total of two factors with an eigenvalue >1 were extracted, accounting for 89.00% of the total variance (Figure 2). Factor 1, accounting for 72.91% of the variance, positively related to Cu, Pb, Zn, Cr, Cd, Na^+^, K^+^ and Cl^−^. The remaining parameters (pH, TDS, Ni, Hg, As, Mg^2+^, Ca^2+^, SO_4_^2−^, HCO_3_^−^, NO_3_^−^ and F^−^) were positively related to factor 2 (16.08%). As a whole, the result of classification based on cluster analysis is consistent with the results from factor analysis.

Many studies have demonstrated that Cu, Pb, Zn, Cr, Cd, Na^+^, K^+^ and Cl^−^ are indicators of roadside contaminated environment [32,33,34,35]. Although the cluster analysis and factor analysis were inadequate to conclude whether the pollutants originated from highway transport activities, the Cu, Pb, Zn, Cr, Cd, Na^+^, K^+^ and Cl^−^ appear to have derived from a similar source, which may be related to traffic. Some studies have shown that highway-related pollutants derived from traffic had a significant negative correlation with distance to the highway [5,6,7], As shown in Table 4, there were significant negative correlations between Cu, Pb, Zn, Cr, Cd, Na^+^, K^+^, Cl^−^ and the distance to the highway through the whole analysis. However, individual indicators did not show significant negative correlations in the different research sites, possibly partly due to the limited number of sampling points. Collectively, the results suggest that Cu, Pb, Zn, Cr, Cd, Na^+^, K^+^, Cl^−^ in the groundwater were related to traffic activities.

### 3.3. The Nonlinear Regression Model of Highway-Related Pollutants

**Figure 3:**
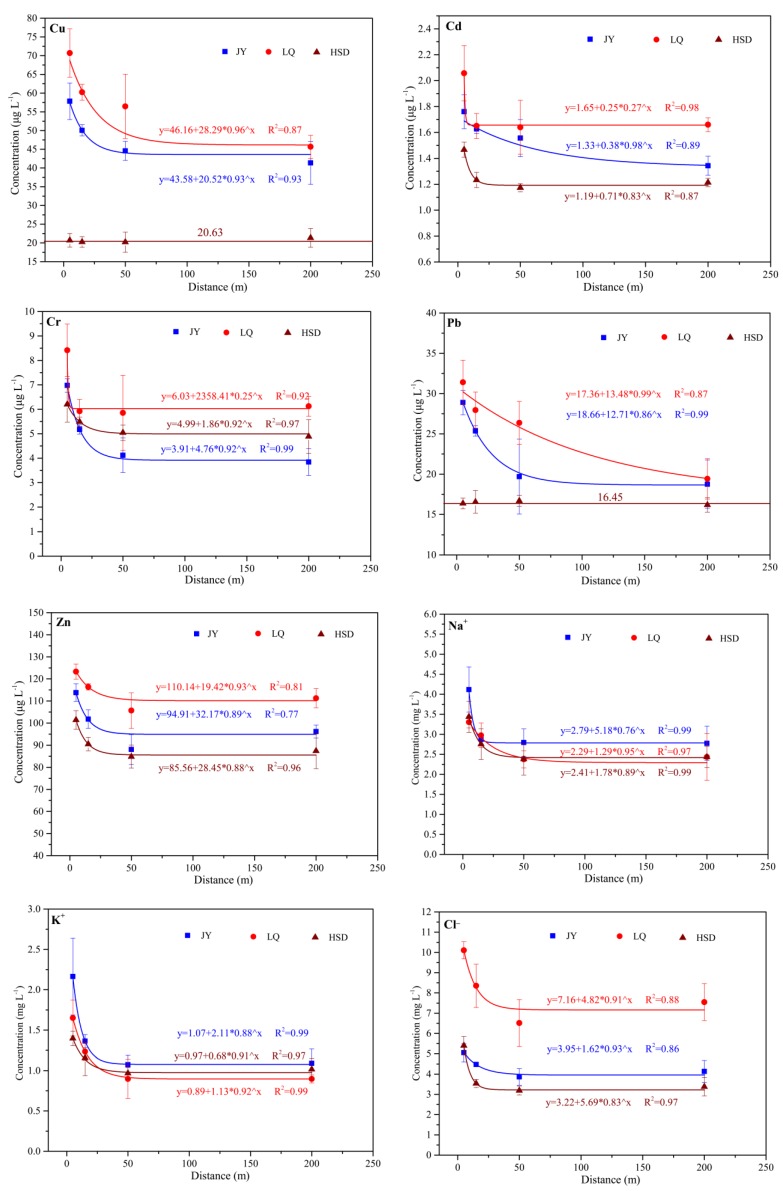
Regression curves of highway-related pollutants concentrations in groundwater in turfy swamp.

The nonlinear regression model (*y* = *a*_0_ + *a*_1_ × *a*_2_*^x^*) was proposed by Wang et al. [36] to describe the distribution characteristics of traffic-related metals contents in turfy swamp soil and plant. As shown in Figure 3, there were two different distribution patterns of the highway-related pollutants in groundwater. The contents of most parameters showed an exponential decline with increasing distance from the road edge in these three sites, whereas the contents of Cu and Pb in HSD site fluctuated with increasing distance from the highway edge. The high R square values showed that this model was also suitable for characterizing the relationship between highway-related pollutants and roadside distance in groundwater. 

The maximum affected distance of highway-related pollutants diffusion is an important parameter for formulating corresponding wetland protection measures. As shown in Figure 3, the contents of Cu, Zn, Cr become the background content at 15 to 50 m distance from the road edge in these three sites. However, the range of maximum affected distance for Pb and Cd varied from 15 to 100 m.

The affected distances of Cu, Zn, Cr, Pb and Cd in groundwater are consistent with those in turfy soil as previously reported by Wang et al. [36]. For Na^+^, K^+^, and Cl^−^, no other study has reported their contents showed an exponential decrease with increasing distance, and reached background contents at 15 to 50 m distance from road edge. Only Earon et al. [12] reported that Na^+^, K^+^ and Cl^−^ concentrations gradually decreased in groundwater with increasing distance from the road edge, but they did not give a specific distribution function.

As shown in Table 5, the background contents of Cu, Pb, Zn, Cr, Cd, Na^+^, K^+^, Cl^−^ at different turfy swamp sites varied, which may be related to the environmental heterogeneity of turfy swamps. In this study, the concentrations of pollutants at all test points were lower than the class III of China Environmental Guidelines, but the concentration of these pollutants in roadside groundwater are obviously higher than the background concentration, which means that the maximum sorption capacity of pollutants is reached and the some pollutants are precipitated from the turfy soil [37,38]. However, this proposal should be interpreted with caution and will require verification during future follow-up studies.

### 3.4. Identification of Metals Pollution Degree in Groundwater

**Figure 4:**
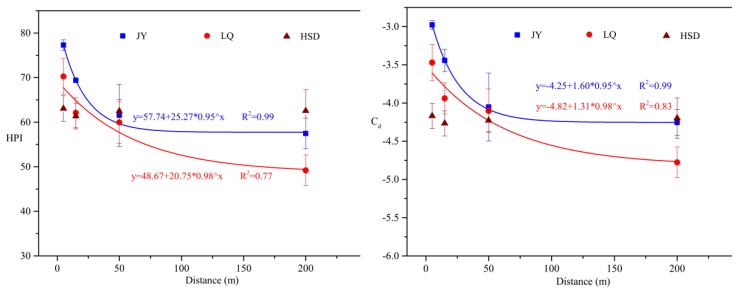
Relationship between HPI and *C_d_* values and distance to the highway edge.

HPI and *C_d_* are effective tools to evaluate the groundwater pollution because they combines several parameters to obtain a particular value which can be compared with the critical value. In this study, the HPI was calculated in different distances from highway using the class III of the China Environmental Guidelines (GB/T 14848-2017), based on Cu, Pb, Zn, Cd, Cr, Ni, Hg and As. The HPI results are shown in Figure 4. The range and mean values of HPI for the groundwater samples in the three sites were 54.1–78.3 and 66.4 (JY), 46.8–72.9 and 60.4 (LQ), and 57.2–66.2 and 62.4 (HSD), respectively. The results showed that the HPIs for all the samples were below the critical limit of 100 proposed for drinking water by Prasad and Bose [39].

The contamination index (*C_d_*) was used as a reference of estimating the degree of metal pollution [40]. The range and mean values of *C_d_* of the groundwater samples were respectively −4.5–−2.9 and −3.7 (JY), −4.9–−3.3 and −4.1 (LQ), and −4.8–−4.4 and −4.7 (HSD). *C_d_* may be grouped into three classes [41] as follows: high (*C_d_* > 3), medium (*C_d_* = 1–3) and low (*C_d_* < 1). In this study, all analyzed samples do not exceed 0, suggesting that they are below the water quality standards, which is consistent with the results of HPI analysis. As reported previously by Wang et al. [28], the degree of metals in turfy soil reached heavily polluted level. In this study, highway-related metals in groundwater are almost no pollution, which may be explained by the strong sorption capacity of turf soil to heavy metals. Many studies have shown that turfy soil has strong potential for heavy metal remediation, and it can effectively reduce heavy metal content in contaminated soil [42,43,44].

In addition, it is worth noting that the values of HPI and *C_d_* decline exponentially with the increase of distance from the highway at these three sites (Figure 4). There are significant negative correlations between the pollution indexes (HPI and *C_d_*) and distance to highway in the JY and LQ site (Table 6). This result can be explained by the lower traffic volume of HSD compared to JY and LQ. In order to investigate the key metals contributing to the computed indices, correlation analysis was performed between the indices (HPI and *C_d_*) and heavy metal concentrations. In JY and LQ, Cu, Pb Zn, Cd and Cr show significant correlations with all the indices, suggesting that these metals are the major contributory parameters (Table 6). For the HSD site, Zn, Cd and Cr are the main contributors to HPI and *C_d_*. Collectively, these results are consistent with the distribution pattern, suggesting that the pollution level is related to traffic activities.

Although the pollution degree of heavy metals in groundwater of turfy swamp is very low, the concentrations of Cu, Pb, Zn, Cr, Cd, Na^+^, K^+^ and Cl^−^ in groundwater near the highway edge are obviously higher than the background concentration values. The correlation analysis between the two pollution index (HPI and *C_d_*) and metals also showed that Cu, Pb, Zn, Cr and Cd are the main contributors to the heavy metal pollution in the groundwater, so it cannot be ignored that operation of highways proximal to turfy swamps may produce heavy metal accumulation in groundwater.

## 4. Conclusions

Nineteen physical and chemical parameters (Cu, Pb, Zn, Cd, Cr, Ni, Hg, As, pH, TDS, Ca^2+^, Mg^2+^, Na^+^, K^+^, SO_4_^2−^, Cl^−^, HCO_3_^−^, NO_3_^−^ and F^−^) were tested in groundwater to evaluate the impact of highway transportation on groundwater environments of proximal turfy swamps. Cu, Pb, Zn, Cr, Cd, Na^+^, K^+^ and Cl^−^ were identified as highway-related pollutants. However, neither the concentrations of pollutants, nor the HPI and *C_d_* indexes, exceeded the water quality standard. The concentration of pollutants were greatest at the edge of the highway followed by an exponential decay away from the highway with the affected distances between 15 to 100 m dependent on location and pollutant. Given the sensitivity and vulnerability of turfy swamps, long-term monitoring of pollution in roadside groundwater is important to establish recommendations for minimal distances of roadways from wetlands.

## Figures and Tables

**Table 1:** Storage requirements and test methods for groundwater samples.

Parameter	Sampling Volume	Container Material	Preservation Method	Test Method
Cu, Pb, Zn, Cd, Ni	500 mL	P	Add concentrated HNO_3_ and adjust pH to 1–2	ICP-MS
Cr	100 mL	P	Add NaOH and adjust pH to 8–9	ICP-MS
Hg	250 mL	B	Add concentrated HNO_3_ and adjust pH to 1–2	Atomic florescence spectrum
As	250 mL	B	Add concentrated H_2_SO_4_ and adjust pH to 1–2	ICP-MS
Ca^2+^, Mg^2+^, Na^+^, K^+^, SO_4_^2−^, Cl^−^, HCO_3_^−^, F^−^	500 mL	P	Original preservation	Ca^2+^, Mg^2+^, Na^+^, K^+^: ICP-AES Cl^−^, F^−^, SO_4_^2−^: Ion chromatography method HCO_3_^−^: Acid-base titration method
NO_3_^−^	100 mL	P	Original preservation	Ion chromatography method

P. Polypropylene bottles; B. Borosilicate bottle; Original preservation: After sampling, no chemical reagents are added.

**Table 2:** Descriptive statistics of heavy metal concentrations (μg L^−1^) in groundwater of turfy swamps.

	Cu	Pb	Zn	Cd	Cr	Hg	Ni	As
JY site								
Mean	48.5a	23.2ab	100.0ab	1.57a	5.0a	0.7b	8.9a	8.7a
SD	7.4	5.0	10.6	0.18	1.4	0.1	1.0	0.8
Min	35.2	15.4	80.3	1.26	3.4	0.5	7.7	7.2
Max	61.5	30.5	118.3	1.91	7.3	0.8	10.8	9.9
CV (%)	15.2	21.5	10.6	11.5	26.8	6.5	8.5	8.6
LQ site								
Mean	58.3a	26.3a	114.2a	1.75a	5.4a	0.08b	6.9b	3.1b
SD	10.5	5.1	8.0	0.22	0.7	0.001	0.8	0.3
Min	43.3	17.6	97.3	1.41	4.23	0.07	5.8	2.4
Max	78.1	34.1	126.8	2.33	6.81	0.08	8.3	3.6
CV (%)	18.1	19.2	17.0	13.1	13.0	3.5	10.8	10.2
HSD site								
Mean	20.6b	16.5b	91.0b	1.27b	6.6a	3.0a	6.9b	8.0a
SD	1.9	0.8	8.1	0.12	1.4	0.1	0.4	0.6
Min	17.3	15.3	79.0	1.14	4.1	2.4	6.3	7.3
Max	23.6	18.1	106.2	1.51	9.2	3.6	7.9	9.7
CV (%)	9.3	5.1	8.9	9.93	21.2	8.3	6.5	7.6
ACV (%)	14.2	15.3	12.2	11.50	20.3	6.1	8.6	8.8
Class III	1000	50	1000	5	50	1	20	10

The data with different superscript lower case letters have significant differences (*p* < 0.05) in the columns; JY. Jingyu site; LQ. Longquan site; HSD. Huangsongdian site; CV. Coefficient of variation; ACV. Average CV; Class III. The water used for the second level reserve of groundwater source for drinking in China.

**Table 3:** Descriptive statistics of hydrochemical parameters concentrations (mg L^−1^) in groundwater of turfy swamps.

	pH	TDS	Ca^2+^	Mg^2^^+^	Na^+^	K^+^	SO_4_^2−^	Cl^−^	HCO_3_^−^	NO_3_^−^	F^−^
JY site											
Mean	5.6	186.8	15.6	7.2	3.1	1.4	12.4	4.4	202.1	0.43	0.11
SD	0.5	3.1	0.9	0.7	0.7	0.5	0.5	0.6	3.1	0.05	0.02
Min	4.4	180.0	14.3	6.5	2.3	0.9	11.9	3.4	195.2	0.37	0.09
Max	6.3	191.0	17.3	8.9	4.6	2.6	13.3	5.6	207.5	0.52	0.15
CV (%)	9.0	1.6	5.9	9.3	21.7	36.2	4.0	13.2	1.6	12.83	9.14
LQ site											
Mean	5.9	271.6	35.7	5.0	2.8	1.2	11.3	8.1	204.8	0.56	0.06
SD	0.7	11.5	2.2	0.6	0.5	0.4	0.7	1.6	4.6	0.03	0.001
Min	4.4	255.1	31.9	4.2	1.8	0.7	10.4	5.4	196.3	0.44	0.04
Max	6.9	296.3	38.3	6.5	3.4	1.9	12.3	10.4	210.6	0.74	0.07
CV (%)	12.5	4.2	6.1	11.9	18.2	30.5	6.2	19.6	2.3	5.01	7.63
HSD site											
Mean	6.2	198.4	36.6	5.2	2.8	1.1	11.1	3.9	132.5	0.33	0.31
SD	0.4	4.2	1.5	0.7	0.5	0.2	0.7	1.0	3.9	0.02	0.01
Min	5.6	189.3	34.1	3.6	1.9	0.9	9.4	3.0	126.5	0.29	0.29
Max	6.7	204.0	38.5	6.0	3.7	1.5	12.3	5.7	138.3	0.37	0.33
CV (%)	6.8	2.1	4.1	9.4	19.5	18.7	6.6	25.1	2.9	7.47	4.35
ACV (%)	9.4	2.7	5.4	10.2	19.8	28.4	5.6	19.3	2.2	8.44	7.04
Total	5.9	218.9	29.3	5.8	2.9	1.2	11.6	5.5	179.8	0.44	0.16
Class III	6.5–8.5	1000	150		200		250	250		20	1.0

JY. Jingyu site; LQ. Longquan site; HSD. Huangsongdian site; CV. Coefficient of variation; ACV. Average CV; Class III. The water used for the second level reserve of groundwater source for drinking in China.

**Table 4:** Pearson correlation coefficients between the affected parameters and distances in groundwater.

Site	Cu	Pb	Zn	Cd	Cr	Na^+^	K^+^	Cl^−^
JY	−0.698 *	−0.666 *	−0.397 *	−0.822 *	−0.671 *	−0.438	−0.528 *	−0.407 *
LQ	−0.797 **	−0.871 **	−0.385	−0.349 *	−0.554	−0.541	−0.606 *	−0.386
HSD	0.208	−0.160	−0.411 *	−0.427	−0.306	−0.488 *	−0.476	−0.450 *
Whole	−0.340 *	−0.440 **	−0.264 *	−0.341 *	−0.416 *	−0.456 **	−0.484 **	−0.288 **

* Correlation is significant at the 0.05 level (two-tailed); ** Correlation is significant at the 0.01 level (two-tailed).

**Table 5:** The background contents of Cu, Pb, Zn, Cr, Cd, Na^+^, K^+^, Cl^−^ in groundwater in different turfy swamp sites.

Site	Cu (μg L^−1^)	Pb (μg L^−1^)	Zn (μg L^−1^)	Cd (μg L^−1^)	Cr (μg L^−1^)	Na^+^ (mg L^−1^)	K^+^ (mg L^−1^)	Cl^−^ (mg L^−1^)
JY	43.6	18.7	94.9	1.3	3.9	2.8	1.1	4.0
LQ	46.2	17.4	110.1	1.7	6.0	2.3	0.9	7.2
HSD	20.6	16.5	85.6	1.2	5.0	2.4	1.0	3.2

**Table 6:** Correlation coefficients for the distance and metal concentrations with indices values.

	JY		LQ		HSD	
	HPI	*C_d_*	HPI	*C_d_*	HPI	*C_d_*
Distance	−0.750 *	−0.739 *	−0.854 **	−0.868 **	0.038	0.047
Cu	0.778 **	0.798 **	0.912 **	0.910 **	0.258	0.221
Pb	0.976 **	0.976 **	0.980 **	0.983 **	0.599 *	0.646 *
Zn	0.660 *	0.666 *	0.621 *	0.620 *	0.213	0.338
Cd	0.718 **	0.694 *	0.642 *	0.623 *	0.739 **	0.608 *
Cr	0.821 **	0.812**	0.621*	0.622 *	0.612 *	0.715 **
Hg	0.203	0.183	0.289	0.282	0.291	0.333
Ni	0.179	0.256	−0.456	−0.460	0.307	0.352
As	0.049	0.082	−0.128	−0.140	0.513	0.562

* Correlation is significant at the 0.05 level (two-tailed); ** Correlation is significant at the 0.01 level (two-tailed).
